# Genotype-Dependent Morphological and Antioxidant Responses of Wild Cherry (*Prunus avium* L.) to Salinity Stress *In Vitro*

**DOI:** 10.3390/plants15091351

**Published:** 2026-04-28

**Authors:** Vanja Vuksanović, Lazar Pavlović, Branislav Kovačević, Marko Kebert, Branislav Trudić, Milica Kovač, Saša Orlović

**Affiliations:** 1 Faculty of Agriculture, University of Novi Sad, Trg Dositeja Obradovića 8, 21000 Novi Sad, Serbia; vanja.vuksanovic@polj.uns.ac.rs (V.V.); milica.kovac@polj.edu.rs (M.K.); sasa.orlovic@polj.uns.ac.rs (S.O.); 2 Institute of Lowland Forestry and Environment, University of Novi Sad, Antona Čehova 13d, 21000 Novi Sad, Serbia; branek@uns.ac.rs (B.K.); kebertm@uns.ac.rs (M.K.); branislav.trudic40@gmail.com (B.T.)

**Keywords:** wild cherry, salt tolerance, tissue culture, phenolics, oxidative stress

## Abstract

Soil salinization is a major abiotic stressor limiting global agricultural and forestry productivity. This study aimed to assess the tolerance of four wild cherry (*Prunus avium* L.) genotypes (8-A, F-12, F-19, F-15) to salinity stress using the *in vitro* culture technique. Shoots were exposed to three NaCl concentrations (0—control treatment, 33, and 100 mM) in micropropagation medium under controlled laboratory conditions for 35 days. Morphological parameters, including shoot length, shoot number, survival and multiplication rate, shoot fresh and dry biomass, and shoot water content, were evaluated alongside biochemical markers such as total phenolic content (TPC), total flavonoid content (TFC), and antioxidant activities assessed through ferric reducing–antioxidant power (FRAP), ABTS radical scavenging, DPPH radical scavenging and nitric oxide (NO•) scavenging. Consistent with the experimental design, exposure to 100 mM NaCl significantly inhibited shoot growth and biomass accumulation, while survival was comparatively less affected. Genotypic variation was evident, with genotypes F-19 and F-12 demonstrating higher tolerance, maintaining greater growth and antioxidant capacity (FRAP and ABTS) under salt stress compared to more sensitive genotypes like 8-A and F-15. Phenolic and flavonoid contents were also reduced at 100 mM NaCl, suggesting that intense salinity stress limited the biosynthesis and accumulation of these antioxidant compounds. Nitric oxide scavenging activity remained largely unaffected by salinity in all genotypes, which may indicate that the applied stress levels were insufficient to markedly alter this component of the antioxidant response. The genotype F-19 emerged as the strongest salinity-tolerant genotype, retaining superior shoot number, multiplication rate, fresh/dry biomass and stable/increased total phenolic content (TPC) under 100 mM NaCl compared to other genotypes. This integrative *in vitro* approach effectively distinguished salt-tolerant wild cherry genotypes and offers a valuable screening tool for breeding and selection programmes targeting improved resilience to salinity stress. The findings have practical relevance for forestry, horticulture, landscape architecture and the restoration of salt-affected sites, particularly in the context of climate change. They also align with current European and global priorities focused on identifying genetically suitable reproductive material for resilient afforestation and ecosystem restoration under increased environmental stress.

## 1. Introduction

Soil salinization represents a significant abiotic stressor that poses substantial challenges to global agricultural productivity. It is estimated that salinity can lead to a reduction in agricultural yields by up to 20% worldwide, underscoring its profound impact on crop production [1]. This issue is particularly pronounced in arid and semi-arid regions, where soil salinization can result in crop yield losses of up to 70% due to the effects of drought and poor water management [2].

The salinization of soil and water resources is further exacerbated by global climate change, which has led to prolonged and severe droughts, forcing farmers to rely on low-quality water sources and unsustainable irrigation practices [3]. Saline soils disrupt plant homeostasis by inducing osmotic stress, reducing water availability as increased salinity lowers the water potential in the soil [4,5]. The most common ions in saline soils include Cl^−^, SO_4_^2−^, HCO_3_^−^, Na^+^, Mg^2+^ and to some extent BO_3_^3−^, which contribute to physiological disorders in plants [4]. These disorders often result from ion toxicity and nutritional imbalances, leading to cell degradation and diminished plant productivity [6]. For instance, the accumulation of Na^+^ and Cl^-^ in plant tissues can cause necrotic shoot tips or leaf margins, further highlighting the detrimental effects of salinity on plant health [7].

Also, salinization is one of the most significant forms of chemical soil degradation in urban environments, often associated with the application of industrial salts for road maintenance during winter months. The use of sodium chloride (NaCl) to melt snow and ice increases soil salt concentrations, negatively impacting the soil’s chemical structure and the plant’s ability to absorb water and nutrients [8]. High concentrations of Na^+^ and Cl^−^ have profound effects on plant survival, growth and development. Among the major impacts of salinity are disruptions in ion homeostasis, which can lead to ion toxicity and nutritional imbalances [9]. Excessive salt concentrations also induce hyperosmotic stress by lowering the water potential in the soil, resulting in reduced or lost turgor pressure and restricted cell expansion [10,11,12]. Additionally, sodium ions disrupt soil aggregates, leading to compaction and decreased porosity, which limits root development and soil aeration [2]. Plants in saline urban soils often exhibit characteristic symptoms, including leaf necrosis, reduced vitality, and growth retardation. Salinization also diminishes ornamental plant value, as sensitive species display necrotic leaf margins and leaf abscission due to chronic salt exposure, degrading the aesthetic quality of urban green spaces [4]. This problem is particularly acute for street trees beside roadsides, where visual appeal is essential [8]. In response to salinity stress, plants often employ adaptive strategies to mitigate these effects. For instance, salinity reduces stomatal aperture and leaf surface area, thereby limiting transpiration and contributing to water conservation under stress conditions [13,14]. This reduction in transpiration helps plants conserve water but can also limit photosynthesis and overall growth. Additionally, high NaCl levels promote the accumulation of sodium and chloride ions in plant tissues. Although these ions play roles in normal physiological processes at low concentrations, their excessive accumulation beyond physiologically optimal levels can exert toxic effects, leading to cellular damage, necrosis, and impaired photosynthetic activity [4,7].

Species genetically adapted to stresses such as salinity and drought, including wild cherry (*Prunus avium*) [15], are vital for maintaining urban greenery in frequently disturbed soils affected by anthropogenic activities.

Wild cherry is a valuable broadleaf species native to Europe, North Africa, and Western Asia, commonly found in mixed forest ecosystems [16]. It thrives in deep, fertile, and moist soils but can establish itself in a wide range of climatic and edaphic conditions. Beyond its role in fruit production, wild cherry is highly valued for its high-quality timber, making it a key resource for the furniture industry [17]. Additionally, it plays a crucial role in agroforestry systems, contributing to sustainable land use and ecological balance [16,18,19]. Wild cherry may be considered a valuable species for nature-based solutions in forestry, agroforestry, and especially in urban environments exposed to abiotic stresses, owing to its ecological and ornamental importance, contribution to ecosystem functioning, and adaptive physiological responses to environmental stress [16,18,19]. The ecological significance of wild cherry extends to its role in maintaining biodiversity, as conserving its genetic diversity is essential for overall ecosystem health [20]. In response to environmental challenges, particularly those associated with climate change, several European countries (Spain, France, Italy, Germany) have initiated breeding programmes aimed at enhancing timber production and tolerance to biotic and abiotic stress factors [21]. These efforts are crucial for ensuring the long-term sustainability and resilience of wild cherry populations.

In addressing these environmental challenges, plant tissue culture emerges as a vital tool. It provides an efficient and controlled environment for studying plant responses to abiotic stresses like salinity and drought [2]. *In vitro* culture allows for the rapid assessment of plant tolerance to various stressors under controlled conditions, bypassing the limitations of field trials, such as the need for large areas and the influence of multifactorial stressors. This approach is particularly beneficial for forest tree species, where traditional breeding methods are time-consuming and resource-intensive due to their long growth cycles [22]. Although salinity stress has been widely studied *in vitro* in several woody plant species (e.g., birch [23], paulownia [24], white poplar [3], citrus [25], *Myrtus communis* [26], pistachio [27]), data on the salinity response of wild cherry (*Prunus avium* L.) remain limited.

Soil salinization represents an increasing ecological constraint in parts of the Western Balkan region and other parts of Europe, particularly in lowland and alluvial areas where groundwater dynamics, irrigation practices, and climate-driven increases in evapotranspiration promote the accumulation of soluble salts in the soil profile [2]. Such conditions can negatively affect the establishment and growth of forest species used in restoration and afforestation programmes. In this context, identifying genotypes with higher tolerance to salinity through controlled *in vitro* screening may support the selection of planting material better adapted to these site conditions. This approach could contribute to more effective forest restoration measures in Europe and the Western Balkans by improving seedling survival, stand establishment, and long-term ecosystem resilience in areas affected by soil salinity.

This study aimed to evaluate the salinity tolerance of selected wild cherry (*Prunus avium* L.) genotypes under controlled *in vitro* conditions. Genotype-specific responses to saline stress were analyzed using morphological (shoot length, number of shoots, survival rate, multiplication rate, fresh and dry weight, water content) and biochemical (total phenolic content (TPC), total flavonoids content (TFC), Ferric Reducing–Antioxidant Power (FRAP) assay, 2,2-difenil-1-pikrilhidrazil (DPPH), 2,2′-azino-bis(3-ethylbenzothiazoline-6-sulfonic acid (ABTS) and Nitric Oxide Radical Scavenging Activity (NO•) indicators to identify variation in tolerance among the tested genotypes.

Within this framework, the study examines whether *in vitro* responses to salinity can support the identification of wild cherry genotype(s) with enhanced salt tolerance and whether *in vitro* culture can be used as an effective early screening approach in woody species selection and breeding particularly in relation to the use of wild cherry in salt-affected environments, including urban and roadside landscapes exposed to deicing salts.

## 2. Results

Analysis of variance showed that NaCl concentration (T) significantly affected most examined traits. Factor genotype (G) also had a strong influence on most of the growth and biochemical parameters (Table 1). The G × T interaction was significant for multiplication rate, water content, TPC, FRAP, ABTS and DPPH, indicating genotype-specific responses to the applied treatments, whereas NO• radical scavenging activity was not significantly affected by any factor.

### 2.1. Morphological Parameters

#### 2.1.1. Shoot Length

**Figure 1:**
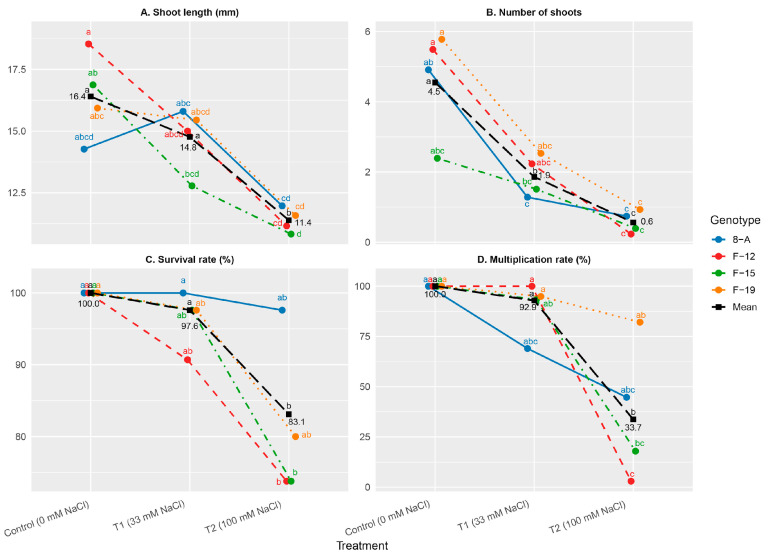
Mean values of: (**A**) shoot length (mm); (**B**) number of shoots; (**C**) survival rate (%); (**D**) multiplication rate (%). Treatments: C: control (0 mM NaCl); T1 (33 mM NaCl); T2 (100 mM NaCl). “Mean” line represents mean values of NaCl concentration treatments. Differences between values of the same trait that are labeled with the same letter are not statistically significant according to Tukey’s HSD test (*p* < 0.05). The test was performed separately at the level of Genotype × NaCl concentration interaction (presented by genotypes) and at the level of NaCl concentration (presented by the “Mean” line).

Although genotypic differences in shoot length were not statistically significant, the applied treatments had a significant effect (*p* < 0.05) on shoot length parameters. The shoot length was approximately 5 mm shorter under the 100 mM NaCl concentration compared to the control. The highest shoot length was recorded in the F-12 control treatment (18.53 mm), followed by F-19 control (15.93 mm) and 8-A T1 (15.80 mm), while the shortest shoots were observed in F-15 T2 (10.83 mm) and F-12 T2 (11.16 mm) (Figure 1A). Among the tested genotypes, genotype F-12 showed the greatest potential for shoot elongation under non-stress conditions, while genotype F-15 exhibited the most pronounced reduction under salt stress. Shoot length was significantly reduced under high salinity (T2, 100 mM NaCl) compared to the control in most genotypes, whereas no significant differences between these treatments were observed in genotypes 8-A and F-19.

#### 2.1.2. Number of Shoots

The number of shoots was significantly affected by genotype and showed the clearest differentiation among the tested NaCl concentrations. A general decline in shoot number was observed with increasing salinity, particularly at 100 mM NaCl. The highest shoot number was observed in the F-19 control treatment (5.78), followed by F-12 (5.49) and 8-A (4.91), although these differences were not statistically significant (Figure 1B). Under high salinity (T2), all genotypes showed a significant reduction in shoot number compared to the control, indicating a strong inhibitory effect of salt stress; however, no statistically significant differences were observed among genotypes within the T2 treatment.

#### 2.1.3. Survival and Multiplication Rate

The survival rate remained high for most treatments, with values above 90% (Figure 1C). However, a slight decrease was observed under the highest salinity level (100 mM NaCl), whose total mean was 83.1%. The survival rate was significantly reduced in all genotypes under high salinity (T2) compared to the control (100%), while no significant differences were detected among genotypes within the T2 treatment. The lowest values were found in F-12 on T2 and F-15 on T2, both showing a survival rate of 73.80%. In contrast, all control treatments exhibited 100% survival.

Multiplication rate was more sensitive to salt stress than survival rate (Figure 1D). While all genotypes under control conditions achieved 100% multiplication, a substantial reduction was observed under high salinity (100 mM NaCl; T2), with a total mean of 33.7%. The decrease was particularly pronounced in genotypes F-12 (3.02%) and F-15 (17.86%). Although genotype F-19 maintained a relatively higher multiplication rate under salt stress (82.14%), overall differences among genotypes under T2 were limited, with statistically significant differences observed only between F-19 and F-12.

### 2.2. Biomass Parameters

#### Biomass Accumulation and Shoot Water Content

**Figure 2:**
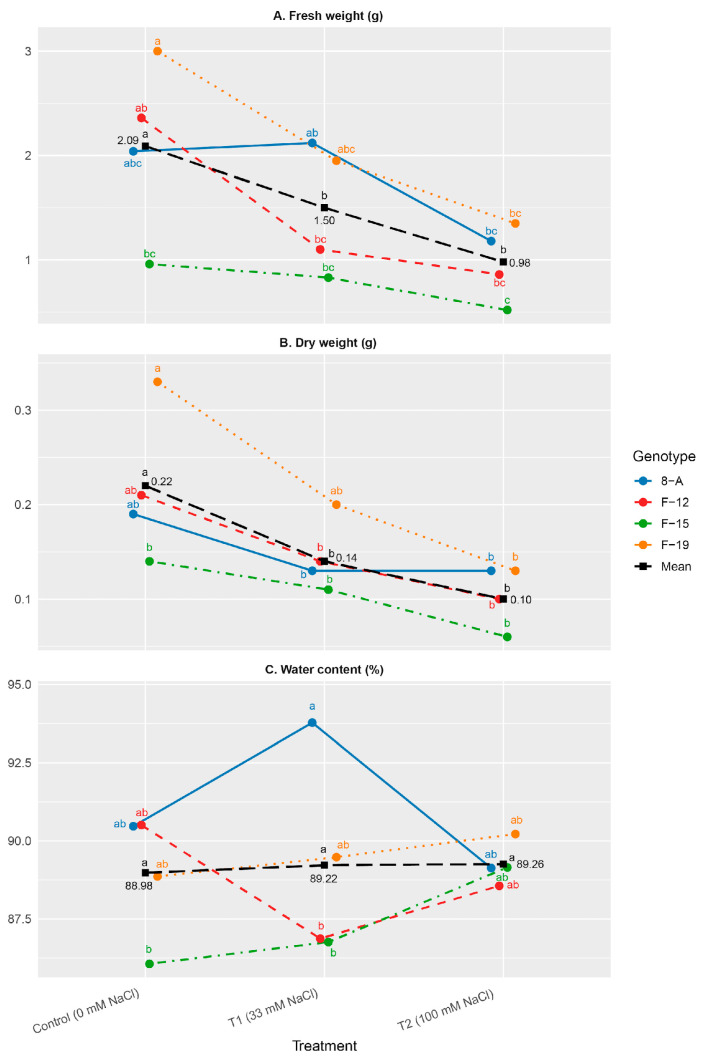
Mean values of: (**A**) fresh weight (g); (**B**) leaf dry weight (g); (**C**) water content (%). Treatments: C: control (0 mM NaCl); T1 (33 mM NaCl); T2 (100 mM NaCl). “Mean” line represents mean values of NaCl concentration treatments. Differences between values of the same trait that are labeled with the same letter are not statistically significant according to Tukey’s HSD test (*p* < 0.05). The test was performed separately at the level of Genotype × NaCl concentration interaction (presented by genotypes) and at the level of NaCl concentration (presented by “Mean” line).

Fresh and dry biomass accumulation, as well as shoot water content, varied significantly across treatments and genotypes. Fresh weight was generally reduced under salinity treatments (T1 and T2) compared to the control.However, a statistically significant reduction in fresh weight was observed only in genotype F-19 under high salinity (T2) compared to the control. Under control conditions, genotype F-19 differed significantly only from F-15, while no significant differences were detected among genotypes under T2. Dry weight under high salinity (T2) was significantly reduced only in genotype F-19 compared to the control, whereas no significant differences between these treatments were observed in genotypes 8-A, F-12, and F-15 (Figure 2B).

Despite fluctuations in biomass, shoot water content remained relatively stable among treatments of NaCl concentration, ranging between 86.06% and 93.78%. The highest water content was recorded in 8-A T1 (93.78%), while the lowest was in F-15 control (86.06%) and F-12 T1 (86.87%) (Figure 2C).

### 2.3. Biochemical Parameters

#### 2.3.1. Total Phenolic Content (TPC) and Flavonoid Content (TFC)

**Figure 3:**
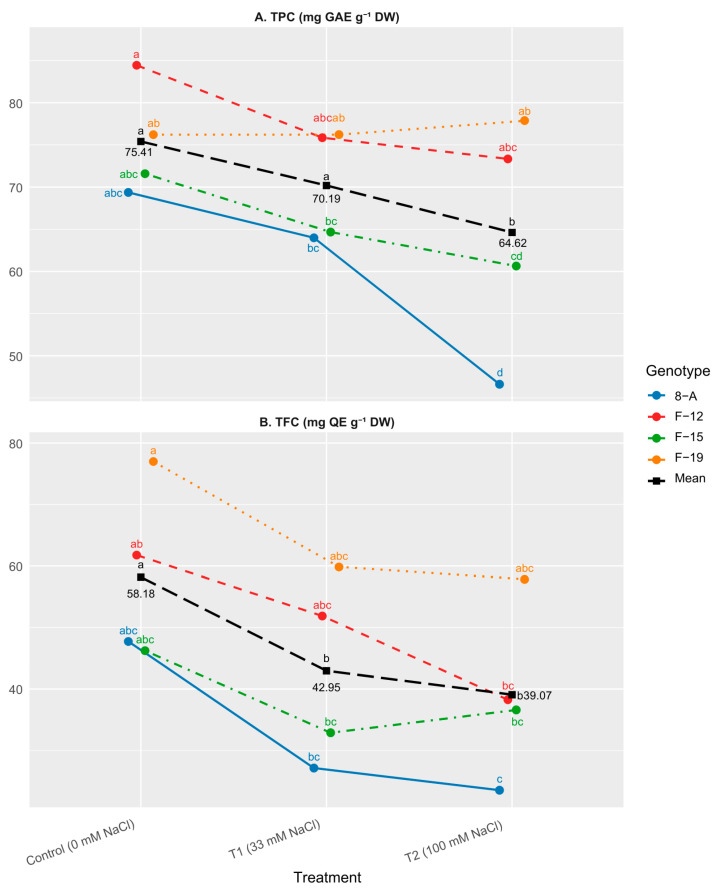
Mean values of: (**A**) total phenolic content (TPC; mg GAE g^−1^ DW); (**B**) total flavonoid content (TFC; mg QE g^−1^ DW). Treatments: C: control (0 mM NaCl); T1 (33 mM NaCl); T2 (100 mM NaCl). “Mean” line represents mean values of NaCl concentration treatments. Differences between values of the same trait that are labeled with the same letter are not statistically significant according to Tukey’s HSD test (*p* < 0.05). The test was performed separately at the level of Genotype × NaCl concentration interaction (presented by genotypes) and at the level of NaCl concentration (presented by “Mean” line).

The highest mean TPC value was observed in genotype F-12 under control conditions (84.5 mg GAE g^−1^ DW), although it did not differ significantly from the other genotypes. Relatively high values were also recorded in genotype F-19 under T2 (77.9 mg GAE g^−1^ DW) (Figure 3A). In total, control conditions resulted in the highest average TPC (75.41 mg GAE g^−1^ DW), while the lowest value was noted under 100 mM NaCl (64.62 mg GAE g^−1^ DW). Only 8-A showed a significant decline in TPC on T2 compared to the control treatment. A statistically significant difference in TPC between genotypes F-12 and 8-A was observed under high salinity (T2), with F-12 showing higher values. Clone F-19 exhibited the highest TPC values under T2 compared to the control, with significantly higher levels than 8-A.

Total flavonoid content (TFC) varied among genotypes and treatments (Figure 3B). High TFC values were observed in genotype F-19 under control conditions (76.99 mg QE g^−1^ DW), as well as in F-12 under control (61.76 mg QE g^−1^ DW) and F-19 under T1 (59.82 mg QE g^−1^ DW), without statistically significant differences among these values. Lower TFC values were recorded in genotype 8-A under T1 (27.19 mg QE g^−1^ DW) and T2 (23.60 mg QE g^−1^ DW), as well as in F-15 under T1 (32.91 mg QE g^−1^ DW) without statistically significant differences among these values. At the genotype level, F-19 showed higher average TFC (64.88 mg QE g^−1^ DW), whereas genotype 8-A exhibited lower values (32.84 mg QE g^−1^ DW), although these differences were not statistically significant across all treatments. Among treatments, the highest average TFC was recorded under control conditions (58.18 mg QE g^−1^ DW), while lower values were observed under T1 (42.95 mg QE g^−1^ DW) and T2 (39.07 mg QE g^−1^ DW).

#### 2.3.2. Ferric Reducing–Antioxidant Power (FRAP) Assay

FRAP values differed significantly among treatments and genotypes, reflecting variations in the antioxidant capacity of the samples. High FRAP activity was observed in genotype F-19 under control conditions (86.72 mM TEAC g^−1^ DW), although it did not differ significantly from genotypes 8-A and F-15, but was significantly higher compared to genotype F-12. At the genotype level, genotypes 8-A and F-19 showed relatively higher average FRAP values (81.97 and 81.38 mM TEAC g^−1^ DW, respectively), whereas genotype F-12 exhibited lower values (70.38 mM TEAC g^−1^ DW), without consistent statistically significant differences among all genotypes. Among treatments, higher mean FRAP activity was observed under control conditions (80.95 mM TEAC g^−1^ DW), while somewhat lower values were recorded under T2 (75.84 mM TEAC g^−1^ DW); however, these differences were not consistently statistically significant.

#### 2.3.3. Radical Scavenging Capacity (RSC)—ABTS Assay

**Figure 4:**
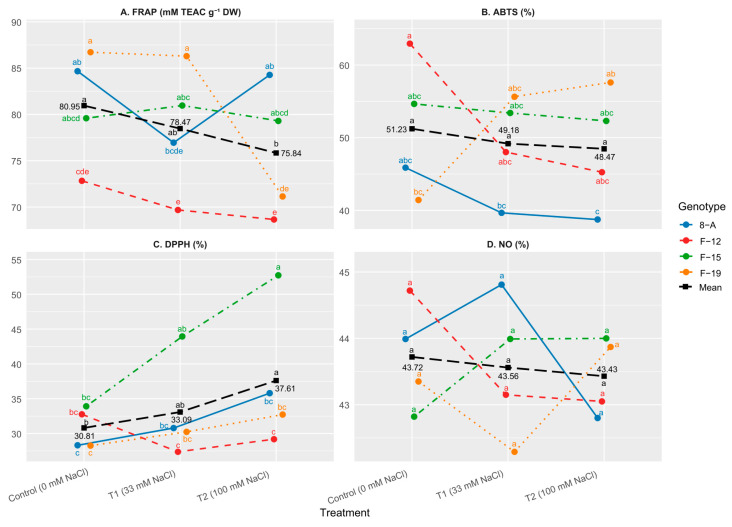
Mean values of: (**A**) Ferric Reducing–Antioxidant Power (FRAP; mM TEAC g^−1^ DW); (**B**) 2,2′-azinobis-3-ethylbenzothiazoline-6-sulfonic acid (ABTS; %); (**C**) 2,2-difenil-1-pikrilhidrazil (DPPH %); (**D**) Nitric Oxide (NO•) Radical Scavenging Activity. Treatments: C: control (0 mM NaCl); T1 (33 mM NaCl); T2 (100 mM NaCl). Differences between values of the same trait that are labeled with the same letter are not statistically significant according to Tukey’s HSD test (*p* < 0.05). The test was performed separately at the level of Genotype × NaCl concentration interaction (presented by genotypes) and at the level of NaCl concentration (presented by “Mean” line).

Radical scavenging capacity (RSC) measured by the ABTS assay varied among genotypes and treatments (Figure 4B). High ABTS values were observed in genotype F-12 under control conditions (62.95%), as well as in genotype F-19 under T2 (57.60%) and T1 (55.64%), without statistically significant differences among several treatments. Lower values were recorded in genotype 8-A under T1 (39.66%) and T2 (38.74%), although these differences were not consistently statistically significant compared to other genotypes. At the genotype level, genotype 8-A exhibited statistically significant lower values (41.42%) compared to F-12, F-15, and F-19. Among treatments, slightly higher mean ABTS activity was observed under control conditions (51.23%), while somewhat lower values were recorded under T2 (48.47%); however, these differences were not consistently statistically significant.

#### 2.3.4. Radical Scavenging Capacity (RSC)—DPPH Assay

DPPH radical scavenging activity varied among genotypes and treatments. A statistically significant increase in DPPH activity under high salinity (T2) compared to the control was observed only in genotype F-15 (Figure 4C). No consistent statistically significant differences were detected among the other genotypes or treatments. 

Radical Scavenging Capacity (RSC) against Nitric Oxide (NO•): The RSC NO• showed minimal variation across treatments and genotypes, with no statistically significant differences observed (*p* > 0.05). Values ranged from 42.29% (F-19 T1) to 44.81% (8-A T1), indicating that this parameter remained relatively stable regardless of salinity level (Figure 4D).

No significant differences were observed for NO• scavenging capacity among genotypes, treatments, or their interaction (*p* > 0.05). 

### 2.4. Correlations Between Traits

**Figure 5:**
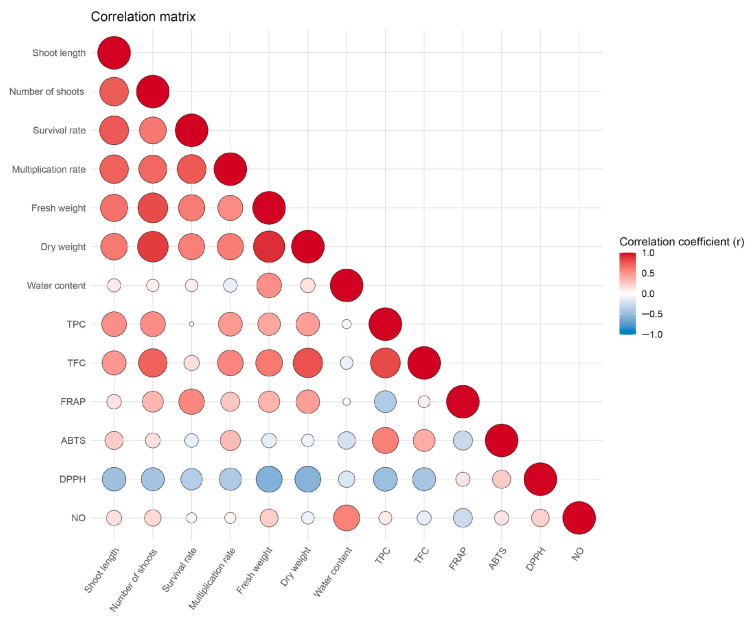
Pearson’s correlation matrix of analyzed parameters.

The correlation analysis revealed significant positive relationships among several morphological and biochemical parameters (Figure 5). Shoot length showed strong positive correlations with the number of shoots (r = 0.73), survival rate (r = 0.75), and multiplication rate (r = 0.72), indicating that longer shoots tend to be associated with higher shoot proliferation and plant viability. Fresh and dry weights were also positively correlated with shoot length (r = 0.65 and r = 0.62, respectively) as well as with the number of shoots, highlighting the linkage between biomass accumulation and shoot growth.

Water content exhibited weak and mostly non-significant correlations with morphological traits but showed a moderate positive correlation with RSC NO• (r = 0.58). TPC and TFC were positively correlated with each other (r = 0.81) and with dry weight (r = 0.45 and 0.78, respectively), indicating that higher biomass is associated with increased accumulation of phenolic compounds. Antioxidant activity assessed by FRAP showed moderate positive correlations with survival rate (r = 0.56) and dry weight (r = 0.45), while ABTS radical scavenging capacity correlated positively with TPC (r = 0.59), supporting the contribution of phenolics to antioxidant potential. In contrast, DPPH radical scavenging capacity was negatively correlated with most morphological and biochemical parameters, including fresh weight (r = –0.58), dry weight (r = –0.58), and total phenolics content (r = –0.48), suggesting the presence of distinct mechanisms or compound profiles influencing DPPH activity. RSC NO• showed weak or non-significant correlations with most variables except for a positive correlation with water content (r = 0.58). The correlation patterns suggest that morphological traits related to shoot growth and biomass formation represent reliable indicators of plant performance under saline conditions. The observed positive relationships among phenolic content, antioxidant capacity, and biomass may indicate that secondary metabolism is involved in the stress response of wild cherry explants. These associations suggest that a combination of growth parameters and selected antioxidant assays may serve as useful indicators for the early screening of salinity tolerance in wild cherry genotypes under *in vitro* conditions.

### 2.5. Principal Component Analysis (PCA)

**Figure 6:**
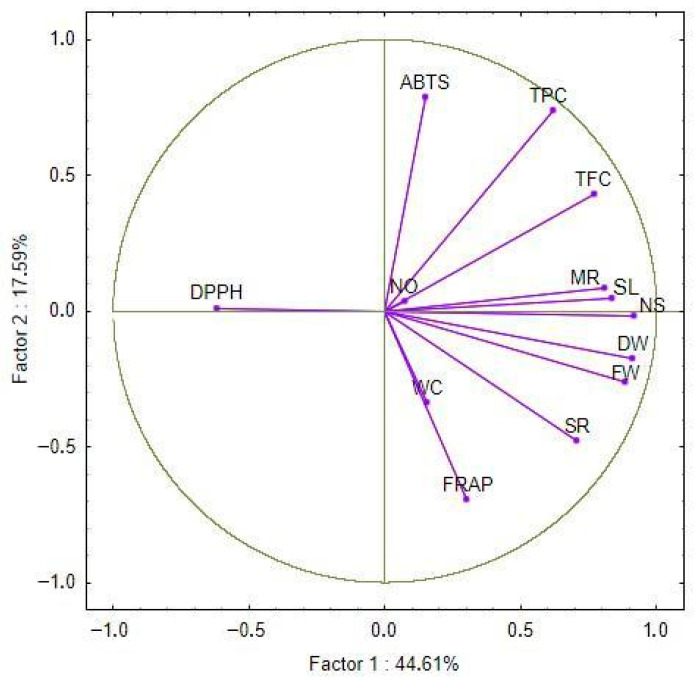
Loadings of the first two principal components for the examined parameters. Abbreviations of examined parameters: shoot length (SL), number of shoots (NS), survival rate (%) (SR), multiplication rate (%) (MR), fresh weight (FW), dry weight (DW), water content (WC), total phenolic content (TPC), total flavonoid content (TFC), Ferric Reducing–Antioxidant Power (FRAP), 2,2′-azinobis-3-ethylbenzothiazoline-6-sulfonic acid (ABTS), 2,2-difenil-1-pikrilhidrazil (DPPH), Nitric Oxide (NO•) Radical Scavenging Activity.

The principal component analysis (PCA) was performed to investigate the relationships among the studied morphological and biochemical traits, as well as to evaluate the relationship between the wild cherry genotypes regarding their reaction with examined salinity treatments. The first two principal components accounted for 44.61% and 17.59% of the total variance, respectively, together explaining 62.20% of the total variance. The loading plot (Figure 6) shows that morphological parameters such as shoot length, number of shoots, multiplication rate, survival rate, fresh weight, and dry weight are positively correlated and cluster closely together along the first principal component (PC1).

**Figure 7:**
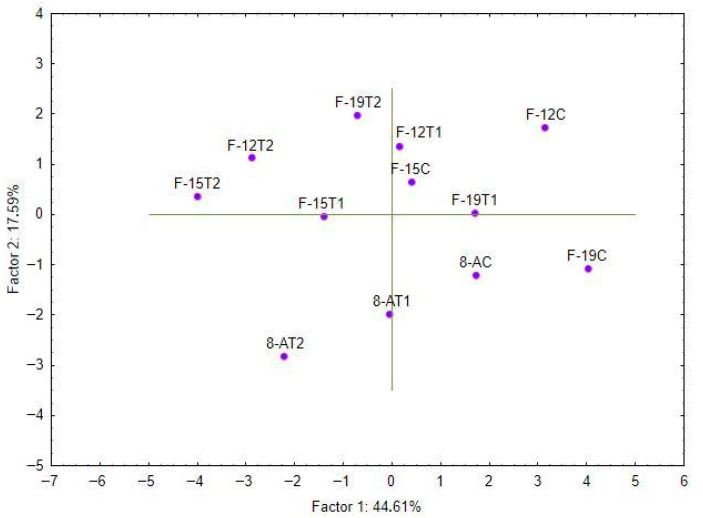
Scores of the first two principal components for the examined treatments at the level of G × T interaction. Abbreviations of examined treatments: The first part of the label stands for the genotype (8-A, F-12, F-15, F-19), and the second part for NaCl concentration (C control—0 mM NaCl); T1 (33 mM NaCl); and T2 (100 mM NaCl).

TPC and TFC also have high loadings with PC1, as well as DPPH radical scavenging activity, which is almost orthogonally positioned against PC2 (meaning that there is poor correlation between them) and negatively correlated with PC1 and previously mentioned parameters. ABTS and FRAP antioxidant activities showed low correlation with PC1 but high loadings on PC2, suggesting that these parameters vary independently of the main morphological and growth-related traits. RSC NO• as well as water content have weak loadings with any of the first two principal components and other traits in general. The score plot (Figure 7) depicts the distribution of reactions of genotypes on NaCl concentrations in the PCA space. Control samples (e.g., 8-AC, F-12C) were generally grouped towards the positive side of the PC1 axis, associated with higher growth and phenolic contents. Treatments with increasing salinity (33 mM and 100 mM NaCl) tend to shift genotypes towards lower PC1 scores, reflecting reduced growth and altered biochemical profiles. There is a clear distinction between genotypes F-12, F-15, and 8-A by PC2, while, opposite to the others, genotype F-19 exhibits greater variation of response to salinity stress by PC2.

## 3. Discussion

Salinity is a major abiotic stress factor that adversely affects plant growth and development by causing osmotic stress and ionic imbalance. Under *in vitro* conditions, where environmental variables are tightly controlled, the effects of salinity on plant morphogenesis and metabolism can be clearly observed [2]. In our study, statistical analysis revealed significant genotype × salinity interactions for key traits, including multiplication rate, total phenolics, and parameters describing antioxidant capacity (FRAP, ABTS, DPPH), demonstrating that wild cherry genotypes employ distinct response strategies under salt stress rather than uniformly scaled reactions. Also, some selected genotypes exposed to 33 and 100 mM NaCl showed a significant reduction in key morphological traits, including shoot length, shoot number, and biomass, particularly under higher salinity levels, whereas the survival rate remained comparatively stable. The same was noticed in biochemical responses of some genotypes that revealed changes in phenolic and flavonoid content as well as in antioxidant activity.

Previous *in vitro* studies on cherry rootstocks have shown similar responses. For instance, Ref. [28] reported that salt stress (50–150 mM NaCl) significantly reduced shoot growth and chlorophyll content in the rootstock Gisela 5, indicating strong growth suppression due to oxidative damage and ion toxicity, though water content remained unaffected. Consistent with these findings, moisture content in shoot tissues in our study remained relatively stable across NaCl treatments. Although salinity lacked a significant main effect on shoot water content, the highly significant genotype × salinity interaction (*p* < 0.05) demonstrates that water balance regulation under salinity stress is genotype-specific, suggesting differential osmotic adjustment or tissue hydration buffering capacities among wild cherry genotypes that link morphological vigor to physiological water homeostasis. Significant reductions in biomass and shoot number were observed, suggesting that salinity stress induced metabolic and developmental constraints that limited resource allocation to growth. A mild increase in shoot number and elongation under 33 mM NaCl was observed in some genotypes (8-A and F-12), which may indicate a potential hormetic response, where low-level salt stress transiently stimulates morphogenesis. Although the differences were not statistically significant, the observed trend is noteworthy and warrants further attention. Similar effects were reported by [29], who demonstrated that 30 mM NaCl promoted shoot growth in *Prunus avium* × *P. pseudocerasus* cv. *Colt*, while higher concentrations (100–150 mM) led to pronounced growth inhibition and leaf damage. Since such effects were not clearly observed in our experiment, further studies including lower NaCl concentrations, as examined in research on accessions of *Populus alba* [3], may help determine whether mild salinity can exert a stimulatory effect on growth. The observed shoot growth reduction can be attributed to the early quiescent phase and growth arrest described in response to salinity. According to [30], salt exposure initiates a temporary cessation of growth driven by ABA accumulation, oxidative signaling, and mechanical cell wall changes, followed by partial recovery in some tissues. In this context, the differences between F-19 and F-15 genotypes suggest distinct capabilities to regulate hormonal and redox balance during early stress perception. Moreover, cell expansion may be restricted due to sodium interference with calcium–pectin cross-linking in the cell wall, as reported in salt-stressed systems. Na^+^ ions can disrupt wall integrity and reduce turgor-dependent elongation, further explaining the sharp reduction in shoot height observed at 100 mM NaCl [31]. The severe growth inhibition observed under 100 mM NaCl in sensitive genotypes is likely associated with disruption of ionic homeostasis and osmotic regulation, since salinity tolerance in plants commonly depends on the maintenance of favorable K^+^/Na^+^ balance and compatible solute accumulation such as proline; these parameters should therefore be included in future mechanistic validation of the most promising genotypes [32].

Exposure to salinity stress often leads to significant alterations in plant secondary metabolism, particularly with regard to the synthesis and accumulation of phenolic compounds and antioxidants, which act as crucial protective agents against oxidative damage [33]. In our study, a genotype-dependent modulation of TPC, TFC, and antioxidant capacity (FRAP, ABTS, DPPH, and RSC NO•) was observed in *Prunus avium* explants subjected to 33 and 100 mM NaCl *in vitro*. A general trend of decreased TPC and TFC under higher salt concentration (100 mM) was evident, particularly in genotype 8-A, while F-12 maintained relatively stable values. These findings are in line with [34], who reported significant reductions in TPC and TFC under 100 mM NaCl in salt-sensitive rice cultivars, while salt-tolerant ones (e.g., Tunca) exhibited increased antioxidant profiles under similar conditions. Likewise, Ref. [3] demonstrated a genotype-dependent decline in phenolic and flavonoid contents in *Populus alba* under 100 mM NaCl, with a concurrent increase in ABTS and DPPH, reflecting a potential activation of the antioxidative defense mechanisms under salinity-related oxidative stress.

Although a slight increase in DPPH and ABTS activity was observed under moderate salt concentration (33 mM) in some cherry genotypes (F-12, F-15, and F-19), the differences were not statistically significant. Nonetheless, previous studies on *Thymus vulgaris* and *Moringa oleifera* reported, besides enhanced activity of antioxidant enzymes (superoxide dismutase (SOD), catalase (CAT), ascorbate peroxidase (APX) and glutathione peroxidase (GPX)), the accumulation of non-enzymatic compounds such as phenolics under salinity stress as well [35,36]. These reports suggest that moderate salinity levels may act as elicitors, promoting the biosynthesis of antioxidant compounds. In contrast, higher salinity levels likely surpass the plant’s adaptive capacity, resulting in oxidative damage and a decline in phenolic production [37,38]. However, such a response pattern was not clearly observed in our study, possibly due to the limited number of tested NaCl concentrations. Further experiments, including a broader gradient of salt levels and additional genotypes, would be required to determine whether moderate salinity can stimulate antioxidant responses in wild cherry. The principal component analysis (PCA) further reinforced these associations, revealing clear clustering of morphological traits (e.g., shoot length, number of shoots, biomass) together with phenolic content and antioxidant parameters such as TFC and DPPH. The divergence of genotypes under different salinity levels, particularly the separation of tolerant genotypes F-19 and F-12 from sensitive 8-A under stress conditions, suggests differential physiological strategies and metabolic plasticity in coping with NaCl-induced stress. This multivariate approach highlights the integrative nature of salinity response and supports PCA as a powerful tool for early genotype discrimination based on complex trait interactions, as demonstrated in tomato [39] and wheat [40] salt tolerance screening. Multivariate analyses reveal a growth-performance cluster (shoot length, number, multiplication rate, fresh/dry weight) positively correlated among themselves but negatively associated with DPPH, while TPC/TFC align more closely with performance retention, confirming that F-19’s salinity resilience stems from preserving propagation/biomass traits with stable biochemical support rather than extreme inducible radical scavenging.

The stable RSC NO• observed across all treatments suggests this pathway may be less sensitive to Na^+^ stress or maintained by compensatory mechanisms, consistent with [41], who reported temporary antioxidant compensation preserving NO• homeostasis in *Arabidopsis thaliana* under short-term salinity. This contrasts with other dynamic antioxidant indices (FRAP, ABTS, DPPH) that exhibited significant genotype × treatment interactions in our study, aligning with broader literature showing salinity-induced modulation of enzymatic/non-enzymatic antioxidants [42]. Both morphological and biochemical responses of *Prunus avium* under *in vitro* salinity stress are highly genotype-dependent. The most pronounced differences among genotypes were observed under strong salinity stress (100 mM NaCl; T2 treatment). While high NaCl concentrations severely inhibit growth and phenolic metabolism in sensitive genotypes, tolerant cultivars such as F-19 and F-12 exhibit more stable performance. The integration of morphological and biochemical assessments in *in vitro* systems may provide a useful framework for the early screening of salt-tolerant cherry genotypes and could support future selection and propagation strategies. Statistical interactions across traits reveal distinct response strategies among wild cherry genotypes rather than uniform tolerance scaling. F-19 emerges as the most robust overall, maintaining a superior shoot number, multiplication rate, biomass, and relatively stable TPC and FRAP under 100 mM NaCl, combining constitutive antioxidant strength with propagation resilience. In contrast, F-12 exhibits trait-specific tolerance with preserved biochemical capacity but multiplication collapses under severe stress; F-15 shows strong inducible DPPH response without morphological benefits; while 8-A represents sensitivity through growth failure and pronounced TPC decline. These multivariate data underscore genotype-specific adaptive strategies beyond simplistic tolerant/sensitive dichotomies.

Relative to comparator taxa, the F-19 genotype shows the closest resemblance to Rootpac R rootstock, which exhibited the most balanced salt tolerance among commercial Prunus rootstocks, as well as to almond genotypes Guara and Penta which maintained favorable growth, physiology, and ion homeostasis under escalating NaCl levels [43]. By contrast, Rootpac 20 displayed only partial tolerance, with shoot elongation offset by severe ion dysregulation and near-complete defoliation. Beyond *Prunus*, *Eucalyptus camaldulensis* demonstrated superior operational robustness compared to intermediates like *Syzygium cumini*, retaining height and biomass while mitigating soil salinity, thus supporting its role in saline reclamation [44]. Among urban woody species under de-icing salt, *Quercus rubra*, *Robinia pseudoacacia*, *Gleditsia triacanthos*, and *Acer campestre* formed a lower-sensitivity group, while *Ginkgo biloba* proved recommendable due to adaptive mitigation despite foliar NaCl accumulation; conversely, *Tilia* × *euchlora* was markedly sensitive and unsuitable for saline roadsides [45,46,47].

The identification of salinity-tolerant genotypes in forest tree species is consistent with current European priorities regarding strengthening the resilience and adaptive capacity of forest genetic resources. European strategies emphasize the importance of identifying and deploying genetically suitable reproductive material that is capable of coping with emerging environmental stresses, including the soil degradation and increasing salinity associated with climate change and anthropogenic pressures. In this context, the early screening of wild cherry genotypes under controlled *in vitro* conditions, as conducted in the present study, represents a useful methodological approach for identifying potentially tolerant genetic material that may support future breeding and restoration efforts [48,49,50]. Such efforts are particularly relevant for forest ecosystem restoration initiatives across Europe and the Western Balkans, where climate change, land degradation, and soil salinization increasingly threaten forest productivity and ecological stability.

A limitation of the present experimental design is the relatively wide gap between the tested salinity levels of 33 and 100 mM NaCl, which restricts interpretation of the transition from mild to severe stress. From a physiological and experimental perspective, 50 mM NaCl appears more appropriate than, e.g., 66 mM NaCl as an additional treatment for future work, because it would better resolve the threshold at which compensatory responses give way to stable growth inhibition. This choice is also ecologically defensible, since salinity is generally considered biologically relevant from approximately 4 dS m^−1^, corresponding to about 40 mM NaCl, making 50 mM a plausible moderate-to-intermediate stress level for screening purposes [51]. This additional concentration would therefore be valuable for distinguishing constitutive tolerance from stress-induced but biologically insufficient responses [52]. Altogether, the inclusion of 50 mM NaCl would improve the dose–response structure of the experiment and strengthen genotype ranking for future acclimation and validation studies. Furthermore, because the experiment was designed as a comparative NaCl-based salinity screening and did not include osmotic control, the relative contributions of osmotic stress and ion-specific toxicity could not be separated.

## 4. Materials and Methods

### 4.1. Plant Material

The study was conducted using four wild cherry (*Prunus avium* L.) genotypes: 8-A, F-12, F-15, and F-19. These four genotypes are accessions from the wild cherry population in the Fruška Gora region (45°9′ N, 19°43′ E) in northern Serbia, selected by vigor, stem form, and overall vitality. The plants were 15 to 20 years old in their adult phase, at the forest edge. The explants were collected from the sun-exposed side of the canopy, 2–4 m above the ground. These genotypes are now part of the genetic collection maintained by the Institute of Lowland Forestry and Environment, University of Novi Sad, Serbia (hereinafter: ILFE). Initial explants were obtained from axillary buds collected during the dormant season in February 2022 to ensure the material was virus-free and genetically stable. These explants were used to establish an *in vitro* culture and further micropropagated to produce material for the establishment of the experiment.

### 4.2. Experimental Design

The experiment was performed in 2023 under sterile conditions in the tissue culture laboratory of the ILFE. Micropropagation was carried out on a modified Murashige and Skoog (MS) medium supplemented with 6.5 g L^−1^ agar, 30 g L^−1^ sucrose, 1 mg L^−1^ benzylaminopurine (BA), 0.5 mg L^−1^ kinetin, 50 mg L^−1^ L-lysine, 50 mg L^−1^ adenine sulfate, and 100 mg L^−1^ myo-inositol. The pH of the medium was adjusted to 5.8 using 1N HCl or 1N NaOH before autoclaving at 121 °C for 20 min. Uniform shoots approximately 2 cm in height were cultured in 25 mL of medium in sterile glass jars, with five explants per sterile jar and three replicates per treatment.

### 4.3. Salinity Treatments

Three salinity treatments were applied: 0 mM NaCl (control), 33 mM NaCl (Treatment 1: T1), and 100 mM NaCl (Treatment 2: T2). These concentrations were selected based on previous studies [2]. Also, these concentrations are supported by field measurements of de-icing salt accumulation in urban and roadside soils, where NaCl levels range from background conditions to moderate (≈30–50 mM, corresponding to electrical conductivity values approaching the 4 dS/m threshold for saline soils) and high (>100 mM in localized hotspots) [53]. Such values are consistent with post-winter increases in soil electrical conductivity, typically 1–5 dS/m (≈10–50 mM NaCl equivalents) in moderately affected areas, and higher in accumulation zones near roads [53,54]. Nevertheless, we acknowledge that inclusion of an additional intermediate concentration such as 50 mM NaCl would have provided a finer dose–response resolution and may be preferable for future screening experiments, which we have described as one of the study’s limitations in the Section 3.

### 4.4. Growth Conditions

Cultures were maintained under controlled conditions for 35 days at 26 ± 2 °C. The photoperiod was set to 16 h of light and 8 h of darkness, with illumination provided by LED lamps emitting white light at an intensity of ~65 µmol m^−2^ s^−1^ PPFD (3500 lux).

### 4.5. Measurement of Morphological Parameters

After 35 days of cultivation, morphological parameters were assessed to evaluate the growth response of the explants. Shoot height (cm) was measured using millimeter graph paper for each plant. The number of newly formed shoots was counted manually and expressed as an absolute number per explant. Survival rate (%) and multiplication rate (%) were calculated at the jar level, based on the number of viable and regenerated explants per jar, with each jar containing five explants. The fresh weight of shoots (g) was determined using an analytical balance. Following fresh weight measurement, plant material was subjected to freeze-drying (lyophilization) for 24 h at −70 °C (modelAlpha 1-2 LDplus, Martin Christ, Osterode am Harz, Germany). After lyophilization, the dry weight of the shoots (g) was recorded. Water content (%) was then calculated as the percentage ratio between fresh and dry weight.

### 4.6. Biochemical Analyses

For the determination of biochemical parameters, fresh plant samples were prepared by homogenizing 20 mg of explants in 2 mL of 96% ethanol. The homogenate was centrifuged at 4000 rpm for 10 min (Microcentrifuge 5424 R, Eppendorf, Hamburg, Germany), after which the supernatant was used for spectrophotometric analysis. All biochemical parameters were determined in 96-well microplates using a MultiScan spectrophotometer (Thermo Fisher Scientific, Multiskan, GO, Waltham, MA, USA).

### 4.7. Total Phenolic Content (TPC)

The total phenolic content was quantified using the Folin–Ciocalteu spectrophotometric (Multiskan GO, Thermo Fisher Scientific, Waltham, MA, USA) method, as described by [55], with gallic acid employed as the standard compound. For the analysis, 25 μL of the ethanolic plant extract was transferred into a microplate, followed by the addition of 125 μL of 0.1 M L^−1^ Folin–Ciocalteu reagent and 100 μL of 7.5% (w/v) sodium carbonate. After the reaction developed, absorbance was measured at 760 nm using a microplate reader. The results were expressed as milligrams of gallic acid equivalent per gram of dry weight (mg GAE g^−1^ DW).

### 4.8. Total Flavonoid Content (TFC)

Total flavonoid content was determined using the aluminum chloride colorimetric method described by [56]. The following reagents were used: 1 M sodium acetate (prepared by dissolving 4.1 g of NaCH_3_COO·3H_2_O in 50 mL of distilled water) and 0.75 M aluminum chloride (prepared by dissolving 5.0025 g of AlCl_3_·3H_2_O in 50 mL of distilled water). In a microplate, 30 μL of plant extract was mixed with 90 μL of methanol, 6 μL of sodium acetate solution, 6 μL of aluminum chloride solution, and 150 μL of distilled water. After 10 min of incubation at room temperature, absorbance was measured at 415 nm using a microplate reader. The flavonoid content was calculated based on a standard calibration curve prepared with quercetin and expressed as milligrams of quercetin equivalent per gram of fresh weight (mg QE g^−1^ FW).

### 4.9. Ferric Reducing–Antioxidant Power (FRAP) Assay

The antioxidant capacity based on ferric ion reduction was determined according to the FRAP assay outlined by [57]. In this method, 225 μL of FRAP reagent, prepared by mixing acetate buffer, TPTZ (2,4,6-tri(2-piridil)-s-triazin) solution, and ferric chloride in a 10:1:1 ratio, was added to 20 μL of ethanolic extract. Following a short incubation period, absorbance was measured at 593 nm. Antioxidant power was expressed as milligrams of ascorbic acid equivalent per gram of dry weight (mg AAE g^−1^ DW), based on a standard calibration curve.

### 4.10. Antioxidant Activity (DPPH and ABTS Assays)

The ability of plant extracts to scavenge free radicals was evaluated using DPPH (2,2-Diphenyl-1-picrylhydrazyl) and ABTS (2,2′-azino-bis(3-ethylbenzothiazoline-6-sulfonic acid) assays, as described by [58,59], respectively. In both assays, the antioxidant potential was determined spectrophotometrically by measuring the decrease in absorbance of the corresponding radical solution after interaction with the sample extract. The reaction mixtures were incubated for 10 min before measurement. Absorbance was recorded at 515 nm for the DPPH assay and at 734 nm for the ABTS assay. The obtained results are expressed as percentages of radical scavenging capacity (RSC). RSC represents the percentage of free radical species neutralized by antioxidants present in the leaf extract. A higher RSC value indicates greater efficiency of the extract in scavenging and neutralizing radicals, and thus reflects a higher antioxidant potential. The RSC (%) was calculated using the following formula:

%RSC = 100 − [(A_A_ − A_B_) × 100/A_C_]

where:

A_A_—absorbance of the tested extract solution;

A_B_—absorbance of the extract dissolved in solvent without DPPH;

A_C_—absorbance of the blank sample containing only DPPH and solvent.

### 4.11. Nitric Oxide (NO•) Radical Scavenging Activity

The inhibition of nitric oxide radicals was assessed using the Griess diazotization reaction, following the method developed by [60]. The procedure involved measuring the absorbance at 546 nm of the colored azo compound formed during the reaction, using a MultiScan GO spectrophotometer (Thermo Scientific, Hamburg, Germany). The degree of NO• inhibition was used as an indicator of antioxidant potential and was expressed as the radical scavenging capacity (RSC).

### 4.12. Data Analysis

The data collected were analyzed using a two-way factorial analysis of variance (ANOVA) to assess the effects of different NaCl concentrations, genotypes, and their interactions. The experiment was arranged in a completely randomized design with three independent jars per genotype × treatment combination. Each jar contained five explants, but the jar was considered the experimental unit in the ANOVA and each replicate was represented by the mean value calculated at jar level. Differences among means were tested by Tukey’s honest significant difference (HSD) test at a significance level of α = 0.05. Relationships between variables were examined using Pearson’s correlation coefficient and principal component analysis (PCA). Principal component analysis (PCA) was conducted using a correlation matrix calculated at the base of mean values of measured variables at the level of genotype × NaCl concentration interaction. Trait relationships were interpreted based on their loadings with the first two principal components that, together, described 62.4% of the total variance. Data processing was carried out with STATISTICA 13 software [61], while graphical visualization was done using R packages ggplot2 [62] and corrplot [63]. Before analysis, the number of new shoots was transformed by a square root transformation (√(x + 1)), and percentage data were transformed using an arcsine transformation (arcsin √x) to meet the normal distribution of frequencies.

## 5. Conclusions

The present study demonstrated that salinity stress significantly affects both morphological and biochemical responses in *Prunus avium* genotypes cultivated under *in vitro* conditions. High NaCl concentration (100 mM) markedly suppressed shoot growth, multiplication, and biomass accumulation, while survival remained relatively stable. Biochemical analyses revealed genotype-dependent variation in phenolic content, flavonoid levels, and antioxidant activity, suggesting differential physiological responses to salinity among the tested genotypes. Genotypes F-12 and F-19 exhibited relatively stable performance across treatments, indicating a potentially higher tolerance to saline-stressed conditions. Correlation analysis and PCA highlighted associations between growth-related parameters and antioxidant activity, suggesting that the integration of morphological and biochemical measurements may be useful for the early screening of salinity responses in wild cherry under controlled conditions. Among the tested genotypes, F-19 should be prioritized in subsequent greenhouse and field-based validation experiments to verify whether its favorable *in vitro* salinity response is maintained under more complex environmental conditions. Moreover, this genotype may serve as a valuable parental candidate for cross-breeding with other genotypes used in this study and others, in future breeding programmes aimed at generating progeny with improved suitability for both *in vitro* and real-time salinity testing.

However, several limitations should be acknowledged. The experiment was conducted under *in vitro* conditions, which do not fully replicate the complexity of field environments where multiple stress factors interact. In addition, only two salinity levels were tested, which limits the ability to detect potential dose-dependent responses or mild stress stimulation effects reported in other plant systems. Therefore, further research involving a wider range of salinity gradients and validation under greenhouse or field conditions will be necessary to confirm the observed patterns and to better assess the applicability of these results for breeding, propagation, or rootstock development under saline environments.

The present findings provide a useful basis for defining the next research and biotechnical steps required to validate, optimize, and operationalize the most promising genotypes for future restoration and planting on salt-affected sites.

## Figures and Tables

**Table 1:** *p*-values from two-way ANOVA for morphological (including biomass) and biochemical parameters.

Trait	Genotype (G)	Treatment (T)	Interaction G × T
Shoot length (mm)	0.355 ^ns^	<0.001 **	0.066 ^ns^
Number of shoots	0.049 *	<0.001 **	0.314 ^ns^
Survival rate (%)	0.036 *	<0.001 **	0.386 ^ns^
Multiplication rate (%)	0.245 ^ns^	<0.001 **	0.027 *
Fresh weight (g)	<0.001 **	0.0001 **	0.206 ^ns^
Dry weight (g)	<0.001 **	0.0001 **	0.401 ^ns^
Water content (%)	<0.001 **	0.931 ^ns^	0.026 *
TPC (mg GAE g^−1^ DW)	<0.001 **	<0.001 **	0.016 *
TFC (mg QE g^−1^ DW)	<0.001 **	<0.001 **	0.887 ^ns^
FRAP (mM TEAC g^−1^ DW)	<0.001 **	<0.001 **	<0.001 **
ABTS (%)	<0.001 **	0.552 ^ns^	<0.001 **
DPPH (%)	<0.001 **	<0.001 **	0.021 *
NO• radical scavenging activity (%)	0.765 ^ns^	0.880 ^ns^	0.226 ^ns^

(*): significant for (*p* < 0.05); (**): significant for (*p* < 0.01), (ns.) not significant; G—Genotype; T—Treatment.

## Data Availability

The raw data supporting the conclusions of this article will be made available by the authors on request.
